# ‘*I think we could probably do more*’: an interview study to explore community pharmacists’ experiences and perspectives of frailty and optimising medicines use in frail older adults

**DOI:** 10.1093/ageing/afae089

**Published:** 2024-05-05

**Authors:** Lucy Faulkner, Carmel M Hughes, Heather E Barry

**Affiliations:** Primary Care Research Group, School of Pharmacy, Queen’s University Belfast, Belfast, UK; Primary Care Research Group, School of Pharmacy, Queen’s University Belfast, Belfast, UK; Primary Care Research Group, School of Pharmacy, Queen’s University Belfast, Belfast, UK

**Keywords:** community pharmacists, frail older adults, medicines optimisation, primary health care, qualitative descriptive research, older people

## Abstract

**Background:**

Community pharmacists potentially have an important role to play in identification of frailty and delivery of interventions to optimise medicines use for frail older adults. However, little is known about their knowledge or views about this role.

**Aim:**

To explore community pharmacists’ knowledge of frailty and assessment, experiences and contact with frail older adults, and perceptions of their role in optimising medicines use for this population.

**Methods:**

Semi-structured interviews conducted between March and December 2020 with 15 community pharmacists in Northern Ireland. Interviews were transcribed verbatim and analysed thematically.

**Results:**

Three broad themes were generated from the data. The first, ‘awareness and understanding of frailty’, highlighted gaps in community pharmacists’ knowledge regarding presentation and identification of frailty and their reluctance to broach potentially challenging conversations with frail older patients. Within the second theme, ‘problem-solving and supporting medication use’, community pharmacists felt a large part of their role was to resolve medicines-related issues for frail older adults through collaboration with other primary healthcare professionals but feedback on the outcome was often not provided upon issue resolution. The third theme, ‘seizing opportunities in primary care to enhance pharmaceutical care provision for frail older adults’, identified areas for further development of the community pharmacist role.

**Conclusions:**

This study has provided an understanding of the views and experiences of community pharmacists about frailty. Community pharmacists’ knowledge deficits about frailty must be addressed and their communication skills enhanced so they may confidently initiate conversations about frailty and medicines use with older adults.

## Key Points

- This study describes community pharmacists’ knowledge and experiences of medicines use and frailty.
- Community pharmacists frequently encounter frail older adults yet highlighted clear deficits in their knowledge and skills.
- Failure to address such issues will hinder community pharmacists’ ability to optimise medicines use for frail older adults.

## Introduction

Frailty is a multidimensional and dynamic condition that can improve or worsen over time, caused by the accumulation of age-related defects across multiple physiological systems [1, 2]. A decline in the body’s in-built reserve results in increased vulnerability following a seemingly innocuous ‘stressor event’, such as infection or medication change [1], negatively impacting quality of life [3] and increasing the risk of adverse outcomes including disability, falls, hospitalisation, institutionalisation and mortality [1, 4, 5]. While frailty is closely related to ageing, it is distinct from any specific disease [2]. The reported prevalence of frailty can vary widely due to different ways in which it can be operationally defined; however, it is higher in older adults, females and nursing home residents [6, 7]. A study by Sezgin and colleagues noted that a prodromal state (pre-frailty) is potentially reversible and may be a target for early intervention [8].

Frail older adults are exposed to greater levels of polypharmacy (commonly defined as use of ≥5 medications) than robust older adults [9, 10], with the former reported to take over six regular medications [11]. Frailty is also more prevalent in older adults receiving polypharmacy [12], with robust older adults receiving polypharmacy reported to have significantly higher odds of developing pre-frailty [13]. Polypharmacy increases the risk of frail older adults receiving potentially inappropriate medications (PIMs) [12], with anticholinergics, antipsychotics, benzodiazepines, non-steroidal anti-inflammatory drugs and sulphonylureas giving cause for concern [9, 10, 14, 15]. Frailty is reported to be associated with medication-related harm, independent of polypharmacy [16]. Non-prescription medication use can also contribute to medication burden in frail and pre-frail older adults and the potential risk for negative outcomes such as adverse drug reactions (ADRs), falls, hospitalisation or other medication-related harm should be considered [17]. It has been suggested that a bi-directional relationship exists between frailty and potentially inappropriate prescribing, with calls for healthcare professionals to monitor older adults for this interaction and intervene when necessary, to minimise patient harm [18]. Increasing medication burden, along with changes in physical function and cognitive abilities, may affect medication administration and adherence, particularly for frail older adults living in their own homes [19, 20]. Medications often continue to be prescribed without thorough review [21], adding to the risk of medication-related harm, yet there is insufficient, high-quality evidence of the effectiveness of medication review for frail older adults [22].

Community pharmacists are one of the most accessible healthcare professionals within primary care, yet their skills are perceived to be underutilised [23, 24]. The need for early identification of frailty, followed by proactive intervention, has been emphasised [25–28]. The research team hypothesised that community pharmacists were likely to have frequent contact with frail older adults due to their medication burden and could play a key role in optimising medication use by this patient population, as highlighted by a recent systematic review [29]. However, little was known about community pharmacists’ knowledge of frailty or their views on how they may contribute to medicines optimisation for frail older adults; previous research had focused on other healthcare professionals and policymakers, and pharmacists were not included [30–32]. Therefore, the aim of this study was to explore community pharmacists’ knowledge of frailty, their experiences and contact with frail older adults, and their perceptions of the role they can play in optimising medicines use for frail older adults.

## Methods

A qualitative descriptive approach was taken [33] whereby data were collected using one-to-one semi-structured interviews conducted with community pharmacists across Northern Ireland (NI). This study is reported in line with the Consolidated criteria for reporting qualitative research (COREQ; Supplementary File 1) [34].

### Participant sampling and recruitment

We sought to recruit 15–20 community pharmacists, as previous studies had indicated this number was sufficient for theoretical data saturation [35]. To achieve this, several sampling and recruitment approaches were used. The first involved Pharmacy Forum NI, the professional leadership body for all pharmacists in NI. Pharmacy Forum NI facilitated sampling through their bi-monthly newsletter, which is emailed to all current registrants of the Pharmaceutical Society of Northern Ireland (PSNI; the regulatory body for pharmacists in NI). A short description of the study was included in the February 2020 newsletter. The second approach involved the Queen’s University Belfast (QUB) School of Pharmacy Undergraduate Placement Network. This comprises 141 ‘Student Training Centre’ pharmacies, which allow undergraduate pharmacy students to undertake placements throughout their degree. Community pharmacies within this network were contacted by email in March 2020 by the network co-ordinator with a short description of the study. As the research team did not have access to the mailing lists used in either sampling approach, a statement was included in the email asking those who had already responded to the piece in the Pharmacy Forum NI newsletter to ignore the invitation. In both approaches, pharmacists interested in participating in the study were directed to a link for a short survey. In this, they were asked to provide information about the length of time they had been working as a community pharmacist, the geographical location and type of community pharmacy in which they worked, and their contact details. The researcher (LF) used this information to purposively sample pharmacists to ensure the resultant sample was diverse and reflected community pharmacists and pharmacies across NI. Those potentially interested in participating in the study were sent an information pack (comprising an invitation letter and information sheet) and followed up after 1 week.

Snowball sampling was also used, where recruited participants were asked to identify other community pharmacist colleagues who may have been interested in participating in the study. This sampling approach proved helpful in recruiting participants from an increasingly hard-to-reach population during the COVID-19 pandemic.

### Data collection

Interviews were conducted by one researcher (LF; a female postgraduate research student trained in qualitative methods) between March and December 2020. The first interview was conducted face-to-face. However, due to COVID-19 pandemic-related restrictions, subsequent interviews were conducted by telephone. The interview topic guide (Supplementary File 2) was developed based on published literature, current frailty guidelines and discussions within the research team to agree content [25–28, 30–32]. Participants were asked to focus on community dwelling frail older adults rather than those living in long-term care settings. Initial questions explored participants’ knowledge of frailty and the health domains they associated with frailty. A definition of frailty was provided, to which participants could refer for the remainder of the interview. Participants’ encounters with community dwelling frail older adults in clinical practice were explored, along with their knowledge and experience of frailty identification within primary care. A clinical scenario involving a fictional frail older patient, ‘Esme’, was presented (Supplementary File 2); participants were asked to reflect on this and their own experiences with community dwelling frail older adults to explore how they would identify and resolve medicines-related issues in this patient population. The topic guide was piloted with three community pharmacists working as academics at QUB; refinements were made prior to commencing data collection. All participants were offered £50 and awarded a certificate of participation. Each interview was audio-recorded with participants’ permission.

### Data analysis

Interview recordings were transcribed verbatim by the researcher; transcripts were de-identified and checked for accuracy. Each participant was assigned a unique identifying code comprising the letters ‘CP’ (community pharmacist) and a two-digit number to indicate the order in which they were interviewed, e.g. CP01. Transcripts were analysed using NVivo 12 Plus [36].

Following an in-depth familiarisation phase that involved listening to the interview audio-recordings, reading and re-reading transcripts, each transcript was independently analysed by two researchers (LF and HB; both qualified pharmacists) using a reflexive thematic analytical approach that was predominantly inductive [37]. The researchers met after coding the first five transcripts to discuss their approaches to analysis and to sense-check emergent codes. Codes were organised into overarching themes around a ‘central organising concept’ [37]; these themes were reviewed and discussed within the research team, during which further refinement occurred until agreement was reached.

### Ethical considerations

This study was approved by QUB Faculty of Medicine, Health & Life Sciences Research Ethics Committee (MHLS 19_15). All participants provided written informed consent.

## Results

Sixteen community pharmacists agreed to participate; one withdrew from the study prior to commencing data collection citing time pressures due to the pandemic. Data saturation was reached after 14 interviews and no further recruitment took place after the 15th interview. On average, interviews lasted 36 min (range: 25–73 min). Participant characteristics are shown in Table 1.

**Table 1:** Characteristics of interview participants (*n* = 15)

**Participant characteristic**	***N***
**Gender** Male Female	10 5
**Number of years of professional experience in community pharmacy** <5 5–9 10–15 >15	5 1 5 4
**Location of community pharmacy** Rural (local population < 5,000) Suburban (population between 5,000–10,000) Urban (population > 10,000)	3 5 7
**Size of pharmacy** Independent (one single premise) Small/medium chain (2–20 individual pharmacy premises) Large chain (>20 linked pharmacy premises)	5 2 8

Three overarching themes were generated from the data and discussed in further detail below.

### Theme 1: awareness and understanding of frailty

Community pharmacists had difficulty defining the term ‘frailty’; while most demonstrated some understanding of the concept through awareness of the frail older adult population and possible signs of frailty, many found it challenging to provide a definition:

*‘It’s* [frailty] *hard to describe, isn’t it? I guess I’d call it someone who’s very vulnerable to illness, breaking bones or just being unwell.’* (CP07).

Most participants focused on physical indicators of frailty based on how they may have observed frail older adults within the pharmacy, with few identifying social or cognitive aspects without further prompting by the interviewer:

“*Obviously in community pharmacy it is all very visual. Maybe mobility, having difficulty coming into the shop, maybe on a three-wheel… rollator*.” (CP03)

*“…I would always associate it with physical strength more than mental capacity.”* (CP11)

Most community pharmacists believed that frailty is an inevitable part of ageing, and few demonstrated an awareness of different levels of frailty. A small number did discuss what they termed as ‘recoverable frailty’ (where patients may be able to transition to a less severe frailty state).

Community pharmacists’ lack of understanding about frailty identification was also highlighted, with most being unaware of assessment tools available:

*“It’s not something I’ve ever thought about, we don’t have any tools readily available to us that I know of and certainly nothing that would be standardised.”* (CP01)

Some community pharmacists reported informally observing older patients and monitoring them for signs of physical or cognitive deterioration:

“*Opening the door is a big one because the door is heavy, so if someone can’t open the door by themselves, I would think about that, looking at how they’re walking, if they’re shuffling…”* (CP06)

However, many participants felt that assessing patients for frailty was not part of their current role and was best left to other primary healthcare professionals, such as general practitioners (GPs). Participants expressed concern regarding the practicality of conducting frailty assessments in a community pharmacy setting, lack of access to patients’ clinical data/medical records and the need for face-to-face contact with frail older adults to conduct the assessments:

*“Why would we need to? We need to be aware of somebody’s frailty but why do we need to initiate the identification or scoring of frailty? …That is something that requires a more collaborative approach.”* (CP10)

Despite this, participants recognised the benefit of knowing a person’s frailty status, highlighting the ways in which having this information to hand could facilitate clinical decision-making and provision of pharmacy services, such as safer provision of non-prescription medicines and checking the clinical appropriateness of prescriptions:

*“You do get a lot of people coming in and asking for co-codamol… and the person standing in front of you could be of average age, absolutely fine but they could be buying it for their 90-year-old mum who weighs 6 stone.”* (CP04)

Community pharmacists felt that the term ‘frail’ had negative connotations and were concerned that patients would not appreciate being labelled as frail and may perceive it as an insult. Furthermore, community pharmacists were not inclined to use the term ‘frail’ when discussing patients with other healthcare professionals or members of the pharmacy team:

*“I probably wouldn’t feel comfortable speaking to the person directly about their frailty, because I would assume it would make them feel old.”* (CP07)

*“There’s a connotation associated with the word* [frail]*… Some older patients may not have physical impairments and may take offence to that if they’re able bodied but have other factors from the definition that would deem them in that category.”* (CP02)

Despite their reluctance to use this terminology with patients, many participants felt that patients would benefit from knowing their frailty status, to improve understanding of their health and empower them to ask for support, if needed:

*“It would be useful for them to know* [their frailty status] *and to recognise where they are in life and what steps they need to take to adjust things.”* (CP05)

Many participants identified the need for increased awareness about frailty both within the pharmacy profession and the general population, further highlighting their own lack of knowledge and need for further training in the area:

*“I think we could probably do more, it’s not something I really recall covering in much detail when we were in university, so… just more education really… I think being more confident, the more that you would study the material and research it.”* (CP15)

*“Community pharmacists need to have a better grasp on what frailty actually is, so they know who to look out for.”* (CP07)

### Theme 2: problem-solving and supporting medication use

Community pharmacists frequently highlighted problem-solving as a key part of their role when dealing with frail older adults and supporting their use of medicines. This was strongly linked with their ability to communicate with a range of relevant stakeholders, such as patients, their carers/family members and other healthcare professionals. Participants emphasised the good relationships they had built with their older patient population, which they believed had been established through consistent and effective communication during previous problem resolution:

*“They* [frail older patients] *also have a better relationship with the pharmacist and are more likely to engage with them because you’ve helped them in the first place.”* (CP05)

While community pharmacists encountered some frail older adults in person in the pharmacy, they regularly communicated with many of their frailest patients either over the telephone or through a carer/family member:

*“During the current climate* [COVID-19 pandemic]*, if a frail older patient needs to see a healthcare professional, they can see one in a community pharmacy. It’s one thing I like about the profession… you might be waiting a couple of minutes, but you can walk in and speak to somebody who is a professional.”* (CP13)

Participants highlighted how they tailored their communication style to individual patients’ needs, for example by slowing their pace of speech or repeating information to ensure patients fully understood:

*“If you know somebody is frail, you’re probably going to have to spend more time relaying information and counselling them, because they may be hard of hearing, they may not understand the first time.”* (CP11)

Despite few participants having identified social aspects of frailty in the earlier part of the interview, many described interacting with older adults due to social isolation or loneliness. Participants regarded themselves as being a point of contact for frail older adults and someone that patients viewed as providing social support:

*“I wouldn’t try and get them off* [the telephone] *as soon as I can because they’ll open up about things and you can be a listening ear for them if they’re feeling lonely.”* (CP15)

In relation to the clinical scenario presented, most pharmacists identified that speaking to the patient directly would be the first step to explore the issues affecting the patient and to build a clearer picture about the patient’s medical and social history so that support could be individually tailored to their needs. Participants also discussed how they communicated with other primary healthcare professionals to resolve medicines-related issues for frail older adults, noting that GPs and GP pharmacists (pharmacists embedded within general practices) as those with whom they would have most frequent contact. Often community pharmacists were in contact with these healthcare professionals to highlight or query potential prescribing issues in frail older adults, express concerns about individual patients, or to arrange further support for vulnerable patients. Participants described how this way of working had led to the development of a shared approach to resolve medicines-related issues in frail older adults:

*“Both of us* [community pharmacist and patient’s GP] *felt we would have an individual conversation with the patient to stress the importance of taking this medication and if she really felt that it wasn’t something that was agreeing with her then we would come up with an alternative.”* (CP10)

However, some participants expressed concern about lack of feedback or action from GPs when potential issues with frail older adults were raised:

*“I would probably give the GP a ring and voice my concern. Now, most of the time that means very little, it means leaving a message at best probably, but I suppose once you’ve done your bit, you must accept that you’ve tried to help in some way.”* (CP06)

Community pharmacists emphasised that supporting and optimising medicines use by frail older adults was a key component of pharmaceutical care provision. They demonstrated extensive knowledge about the medicines-related issues faced by this patient population, such as inappropriate prescribing (e.g. use of PIMs or high-risk medicines) and polypharmacy, unclear medication indications, increased risk of adverse drug effects and medication non-adherence:

*“Can we establish any sort of issues that they’re* [Esme – clinical scenario patient] *having with their medications? Any reason why they don’t want to take them or any side-effects that are impacting their daily life… contributing to their frailty... the likes of any drowsy medication… do they really need that medication or is there an alternative that would benefit them without having that side-effect?”* (CP08)

Community pharmacists described having a vital role in assisting frail older patients with prescription ordering and acquisition, dispensing medication in weekly compliance aids, and providing home delivery services and considered these strategies important in reducing patients’ social isolation and negative outcomes such as falls and hospitalisation.

Participants viewed themselves as a key point of contact for frail older adults and their carers/family members, highlighting that their position within primary care enabled them to either assist patients personally or signpost them to relevant services, organisations or other sources of information/support. Indeed, signposting was identified as a key aspect of ‘problem-solving’ for frail older adults in primary care:

*“People come in all the time looking for solutions and half the time you’re thinking how can I help this person, what do I know, how can I point them in the right direction. We’re problem solvers.”* (CP10)

Although participants felt that signposting had a positive impact for frail older adults and stressed the importance of multidisciplinary support for these patients, they were often unaware of the outcome, again reporting that there was little or no feedback from GPs or other healthcare professionals.

### Theme 3: seizing opportunities in primary care to enhance pharmaceutical care provision for frail older adults

Participants identified opportunities within primary care to improve the management of frail older adults and their medicines use, primarily with respect to pharmaceutical care provision. Many participants felt that the community pharmacist role needed to continue to evolve beyond dispensing and medication supply, highlighting that if they were not independent prescribers this limited their influence over patients’ medications as they had to involve other healthcare professionals to make amendments to medications, when necessary:

“*I think that pharmacists in primary care, both in community pharmacy and in GP surgeries, that are limited by their inability to prescribe* [if not qualified independent prescribers]*… that should be advanced for more pharmacists*.” (CP13)

Participants also indicated that ‘getting out of the dispensary’ would be key to assisting frail older adults with their medicines. Community pharmacists explained that their frailest patients were often housebound and struggled to present in person to the pharmacy. Home visits would improve pharmacists’ understanding of how frail older adults store and use their medication so that tailored support and solutions could be offered to these patients. However, pharmacists stated they were often hindered in making home visits due to lack of funding, time and pharmacist cover:

*“You could go out to the patient’s house* [to conduct medication review] *because you see exactly how they’re managing their medicines. They’ve had them in bizarre places… all you see are Tupperware® boxes* [plastic or glass food storage containers with snap close lids] *all over the house, you can definitely see a true picture that way… you could speak to them over the ‘phone too, but face-to-face is better.”* (CP15)

Participants identified several barriers to seizing opportunities in primary care to enhance pharmaceutical care provision for frail older adults including lack of resources such as time, a private consultation room, support staff and excessive paperwork. The COVID-19 pandemic, ongoing at the time of data collection, was also at the forefront of participants’ minds:

*“Increased workload, reduced staff… at the moment COVID-19 is the big one… but after that it will always come down to time pressure because patient safety has to be the number one priority.”* (CP01)

Community pharmacists believed lack of knowledge around the frail older population was another barrier to delivering high quality pharmaceutical care to frail older adults and further training was needed for both pharmacists and support staff; some indicated they intended to undertake additional training on frailty for their continuing professional development (CPD) because of their participation in the study:

*“I think with you highlighting this… I need to do a bit of CPD... and think about what I could do for frail patients.”* (CP04)

Participants perceived community pharmacy to be isolated within primary care with respect to information sharing, with restricted access to patients’ clinical data, medical or medication histories other than medication data from prescriptions that had previously been dispensed in that pharmacy. Participants believed this placed them at a disadvantage compared with other healthcare professionals, such as GPs, GP pharmacists or colleagues in secondary care and impeded medicines optimisation activities in community pharmacy, such as medication review:

“*I do feel that community pharmacy is in the dark… we don’t see bloods, we only see what we dispense, so if you have a patient that’s going to other pharmacies, I have no idea what else they could be on...”* (CP03)

“Y*ou’re only working off what you have in front of you on your PMR* [patient medication record] … *you can make recommendations, but the GP surgery can overrule you because they know the situation better.”* (CP12)

## Discussion

This qualitative study is the first to explore community pharmacists’ knowledge of frailty among community dwelling older adults, their contact with frail older adults and their perceptions of the role they can play in optimising medicines use in this patient population. It has therefore addressed vital gaps in the current evidence base and provided novel insights into the views and experiences of these healthcare professionals.

The findings have highlighted that community pharmacists regularly encounter frail older adults in their clinical practice and yet there was a significant gap in their knowledge about frailty, with many struggling to define the term and focusing solely on physical signs. Research conducted with other healthcare professionals supports this finding [38–40]. Indeed, Aygerinou and colleagues identified a ‘gap in geriatric education’ as a barrier to identifying and managing frailty in primary care, with most of their study participants (physicians, nurses and health visitors working in primary care) having received no undergraduate or postgraduate training in geriatrics [39]. Many of our study participants also identified that further education in frailty is essential to improve and extend pharmaceutical care provision to frail older adults in community pharmacy. None of our participants had completed frailty-specific training but some identified an opportunity to focus on this as part of their professional development. While several pharmacists had completed postgraduate training in managing medicines in older people, courses such as these do not provide extensive detail about frailty and take a more generalised approach when considering older adults. Our findings suggest that pharmacists would welcome an educational programme, especially if it was specific to the area of interest and tailored to the community pharmacy setting [41]. A systematic review conducted in 2018, which set out to critically appraise studies that investigated the effectiveness of comprehensive educational programmes for healthcare professionals related to frailty prevention or management [42], found no studies to include in the review, which further emphasises the lack of educational programmes addressing this topic.

None of the interview participants were assessing patients for frailty in their routine practice. A lack of awareness of frailty assessment tools or how they could be implemented in the community pharmacy setting was demonstrated. The British Geriatrics Society recommends that older people should be assessed for frailty during all encounters with health and social care professionals [26], including community pharmacists. Rhalimi and colleagues explored the role of community pharmacists in detecting the prevalence of frailty and spatio-temporal disorientation among community dwelling older adults [43]. They concluded it was feasible for community pharmacists to perform this screening and stated that knowledge of older adults’ frailty status was useful for community pharmacists as they may be able to detect more ADRs [43]. Many pharmacists in our study also felt that having knowledge about a patient’s frailty status would be beneficial to them and the patient. Yet, our participants were reluctant to discuss frailty with older adults due to negative connotations associated with the term and resultant challenges such conversations would bring. Studies conducted with other healthcare professionals have also revealed similar views [31, 40], with acknowledgement that training is needed across both primary and secondary care to overcome stigma associated with this terminology. Qualitative studies undertaken with older adults regarding their perceptions of frailty have reported that they rejected the term or did not identify as such [44, 45] and recommended that healthcare professionals instead focus on patients’ independence, resilience and autonomy [45].

Although the community pharmacists in our study were reluctant to discuss frailty with patients, many felt it was important for frail older adults to be aware of their frailty status. Having a person-centred focus and ensuring patients have sufficient knowledge regarding their health and medical conditions are key facilitators for shared decision-making [46]. Effective communication between healthcare professionals and patients must be established so that frail older adults can be empowered and emboldened to make informed decisions about their care. Lawless and colleagues highlighted the need for ‘specialised communication skills training programmes’ which could facilitate timely conversations with frail older adults and allow primary healthcare professionals to provide practical support and guidance to patients [47].

Community pharmacist participants felt they could play a more significant role in optimising medicines use for frail older adults. Interestingly, much of the previously published work in this area had not included community pharmacists. Policymakers and service commissioners must be cognisant of the frequent contact and trusting relationships that community pharmacists have with this patient population [48] and work to address some of the barriers identified in this study, such as the lack of access to patients’ medical histories and clinical data, which isolate community pharmacists from their counterparts in other areas of primary and secondary care.

### Strengths and limitations

This study makes an original and important contribution to the frailty literature. Taking a qualitative approach ensured that participants’ views and experiences could be explored in-depth. Several sampling approaches were used to ensure diversity in the participant sample with respect to their experience in community pharmacy and characteristics of the pharmacies in which they worked. This study took place during the early months of the COVID-19 pandemic, a time during which community pharmacists were playing a critical role in the public health response and experiencing unprecedented challenges due to increased workload and staff shortages [49, 50]. Despite this, the study was able to continue, albeit with minor amendments to the mode of data collection and achieve data saturation. However, the findings only reflect the views of those who participated in the study (the participant sample included a very small proportion of all community pharmacists in NI and had more males than females) and may not be generalisable to other areas of the UK or healthcare settings in other countries. We acknowledge that sampling pharmacists through the Pharmacy Forum NI newsletter and Undergraduate Placement Network may have attracted pharmacists who were more engaged in the topic area and/or research. The use of snowball sampling may have limited the diversity of the participant sample; however, we would have been unable to reach data saturation without using this sampling approach. Due to time constraints on the project, it was not possible to undertake member validation. A reflexive approach was followed throughout the study, with the research team maintaining an awareness of how their personal positioning, experiences or beliefs may have impacted their observations, interpretations or conclusions. All interviews were conducted by a female pharmacist; while this may be considered a strength due to her ability to build a rapport with participants, facilitate an open discussion and understand the context relating to the issues discussed, it should be acknowledged that participants’ awareness of her professional background may have affected their responses. Participants in this study were asked to focus on community dwelling frail older adults; we acknowledge that the findings likely relate to patients with mild–moderate levels of frailty and approaches to support those with more advanced frailty may differ. We recognise that community pharmacists only represent one key stakeholder in the primary care setting. While it was outside the scope of this study to interview other healthcare professionals, patients or carers, further work is needed to corroborate the findings and explore the perspectives of other stakeholder groups.

## Conclusion

Community pharmacists have frequent contact with frail older adults and resolve a range of medicines-related problems for this patient population. However, they have identified distinct gaps in their knowledge about frailty and its identification and hesitancies around initiating conversations with frail older adults about frailty. They also identified system-level barriers, which were perceived to isolate them from other areas of primary and secondary care. These issues must be addressed so that community pharmacists can play a more significant role in optimising medicines use for frail older adults in the future.

## Supplementary Material

aa-23-1962-File002_afae089
