# Supplementary material for: ‘I think we could probably do more’: an interview study to explore community pharmacists’ experiences and perspectives of frailty and optimising medicines use in frail older adults
Source: Age Ageing. 2024 May 5;53(5):afae089. doi: 10.1093/ageing/afae089 (PMC11070721; doi:10.1093/ageing/afae089)
Supplement: aa-23-1962-File002_afae089 [file aa-23-1962-file002_afae089.docx]

**“*I think we could probably do more*”: An interview study to explore community pharmacists’ experiences and perspectives of frailty and optimising medicines use in frail older adults.**

**List of contents**

| **Supplementary file 1** | COREQ (Consolidated criteria for Reporting Qualitative research) checklist (Tong et al., 2007) |
| --- | --- |
| **Supplementary file 2** | Interview topic guide |

**Supplementary file 1**. COREQ (Consolidated criteria for Reporting Qualitative research) checklist (Tong *et al*., 2007)

| **Topic** | **Item No.** | **Guide Questions/Description** | **Description and manuscript page number (where relevant)** |
| --- | --- | --- | --- |
| **Domain 1: Research team and reflexivity** | | | |
| *Personal characteristics* | | | |
| Interviewer/facilitator | 1 | Which author/s conducted the interview or focus group? | Interviews were conducted by Lucy Faulkner (LF; page 6) |
| Credentials | 2 | What were the researcher’s credentials? E.g. PhD, MD | LF had an MPharm degree and was a PhD candidate at the time of data collection (pages 6 and 7). |
| Occupation | 3 | What was their occupation at the time of the study? | LF was a PhD student at the time of the study (page 6). LF continued to practise pharmacy part-time in the community setting. |
| Gender | 4 | Was the researcher male or female? | Female (page 6). |
| Experience and training | 5 | What experience or training did the researcher have? | LF had undergone training in qualitative research methods (page 6). The study was over seen by HB (the study CI) who had previous qualitative experience. |
| *Relationship with participants* | | | |
| Relationship established | 6 | Was a relationship established prior to study commencement? | Some participants were known to LF from working in community pharmacy. It is acknowledged that participants’ awareness of LF’s professional background may have affected their responses (page 21). |
| Participant knowledge of the interviewer | 7 | What did the participants know about the researcher? e.g. personal goals, reasons for doing the research | Participants were aware that LF was a PhD student, working at QUB School of Pharmacy at the time of the study. Participants were given an overview of the study before the interview commenced (Supplementary file 2) as well as a participant information sheet (page 6) to refer to as needed. |
| Interviewer characteristics | 8 | What characteristics were reported about the interviewer/ facilitator? e.g. Bias, assumptions, reasons, and interests in the research topic | LF had an interest in the research topic of frailty and medicines optimisation. LF also had an interest in research involving community pharmacists. |
| **Domain 2: Study design** | | | |
| *Theoretical framework* | | | |
| Methodological orientation and Theory | 9 | What methodological orientation was stated to underpin the study? e.g. grounded theory, discourse analysis, ethnography, phenomenology, content analysis | The interview topic guide was developed using published literature, current frailty guidelines and following discussions within the research team. Thematic analysis was conducted on all transcripts by two researchers (LF and HB; page 7). |
| *Participant selection* | | | |
| Sampling | 10 | How were participants selected? e.g. purposive, convenience, consecutive, snowball | Purposive sampling was used to select participants sampled through the Pharmacy Forum NI newsletter and the QUB undergraduate placement network. Snowball sampling was then utilised (pages 5-6). |
| Method of approach | 11 | How were participants approached? e.g. face-to-face, telephone, mail, email | Participants who expressed interest in the study were initially contacted by the researcher (LF) using email addresses they provided (pages 5-6). |
| Sample size | 12 | How many participants were in the study? | Fifteen participants (page 8). |
| Non-participation | 13 | How many people refused to participate or dropped out? Reasons? | One participant withdrew due to COVID-19 related time constraints (page 8). |
| *Setting* | | | |
| Setting of data collection | 14 | Where was the data collected? e.g. home, clinic, workplace | One interview was conducted face-to-face at a venue selected by the participant. Due to the COVID-19 pandemic, subsequent interviews took place via the telephone (page 6). |
| Presence of non-participants | 15 | Was anyone else present besides the participants and researchers? | To the best of LF’s knowledge non-participants were not present during data collection. |
| Description of sample | 16 | What are the important characteristics of the sample? e.g. demographic data, date | Participant characteristics are presented in Table 1 (page 27). |
| *Data collection* | | | |
| Interview guide | 17 | Were questions, prompts, guides provided by the authors? Was it pilot tested? | The research team developed a semi-structured topic guide with questions and prompts (Supplementary file 2). which was piloted and refined prior to use (page 7). |
| Repeat interviews | 18 | Were repeat interviews carried out? If yes, how many? | No repeat interviews were needed. |
| Audio/visual recording | 19 | Did the research use audio or visual recording to collect the data? | All interviews were audio-recorded (page 7). |
| Field notes | 20 | Were field notes made during and/or after the interview or focus group? | No field notes were made by LF during the interviews. All audio recordings and interview transcriptions were checked to ensure all information had been captured (page 7). |
| Duration | 21 | What was the duration of the interviews or focus group? | Interviews lasted between 25 and 73 minutes (page 8). |
| Data saturation | 22 | Was data saturation discussed? | Data saturation was reached after 14 interviews (page 8). |
| Transcripts returned | 23 | Were transcripts returned to participants for comment and/or | Transcripts were not returned to study participants for comment, due to time constraints on the study (acknowledged as limitation; page 21). |
| **Domain 3: analysis and findings** | | | |
| Data analysis | | | |
| Number of data coders | 24 | How many data coders coded the data? | Two researchers (LF and HB; page 7). |
| Description of the coding tree | 25 | Did authors provide a description of the coding tree? | No, although this can be provided upon reasonable request. |
| Derivation of themes | 26 | Were themes identified in advance or derived from the data? | An inductive analytical approach was used where themes were derived from the data (page 7). |
| Software | 27 | What software, if applicable, was used to manage the data? | NVivo® version 12 software (page 7). |
| Participant checking | 28 | Did participants provide feedback on the findings? | Due to time constraints on the study this was not done (acknowledged as limitation; page 21). |
| Reporting | | | |
| Quotations presented | 29 | Were participant quotations presented to illustrate the themes / findings? Was each quotation identified? *e.g. participant number* | Illustrative quotations have been presented; each quotation is identifiable through the anonymous code assigned to each participant (pages 8-17). |
| Data and findings consistent | 30 | Was there consistency between the data presented and the findings? | Yes, data presented on pages 8-17 are consistent with the discussion presented on pages 17-22. |
| Clarity of major themes | 31 | Were major themes clearly presented in the findings? | Yes, three overarching themes were generated from the data (pages 8-17). |
| Clarity of minor themes | 32 | Is there a description of diverse cases or discussion of minor themes? | Yes (pages 8-17). |

**Supplementary file 2:** Interview topic guide

**Community Pharmacist Interview Topic Guide**

An exploration of community pharmacists’ experience, knowledge and perspectives of frailty and medicines optimisation in frail older patients: A qualitative study.

**Introduction**

“*My name is Lucy Faulkner, and I am a PhD student from the School of Pharmacy, Queen’s University Belfast. Thank you very much for making the time to speak with me today.*

*In this research project, we are interested in exploring community pharmacists’ perspectives on medicines optimisation in frail older people. We plan to analyse the information we gather during this study, which will inform the development of a future questionnaire for distribution to all community pharmacists across Northern Ireland. Have you had a chance to read through the information sheet that was sent out to you? Are there any questions that you would like to ask me before we start?*

**Explaining what will happen in the interview and afterwards**

*“The aim of this interview is to explore your knowledge and awareness of frailty, your experiences with frail older people, how medicines use can be optimised for frail older people, and your perceptions of the barriers and facilitators to successful medicines optimisation for older frail people living in the community. We are focusing on people with frailty living within the community setting rather than a nursing or residential care home facility, so you should bear this in mind as you answer the questions during the interview. The interview should last approximately 40 minutes.*

*I will be recording this interview using a digital recorder, to ensure that there is an accurate and detailed record of what you say. The interview recording will be typed up word-for-word and any names, locations, or anything else that could possibly identify you will be removed so that you remain anonymous. Once transcribing is completed and checked, the original audio recording will be deleted. After interviews have been completed with all of the other participants, the information will be analysed within the research team. Transcripts will only be read by those directly involved in the research study and will be saved on the secure University server.*

*You are free to stop the interview and/or the recording at any point. If, during the interview, there are any questions you would rather not answer, please let me know and we can move on to the next question.*

*Before we begin, I need you to provide written consent that you understand what the interview involves; that anything you say will be kept strictly confidential; you will not be identified in any way; you can stop this interview at any time; and that you are happy for the interview to be recorded. Can you read through the consent form and initial each box to indicate that you understand and agree with each statement please? There are two copies of the consent form. I need you complete and sign both copies: you will keep one and I will keep the other for our records.*

*Have you any further questions about the study before we start the interview?”*

[Turn the digital recorder on]

**Background information**

- Can you tell me how long you have been practising as a community pharmacist?
- Do you hold any postgraduate clinical qualifications, such as a certificate in non-medical prescribing?
- Have you completed any additional training (either formal or self-directed) in the areas of medicines optimisation or frailty?

**Understanding of frailty and frailty identification**

*“I’d now like to ask you about the term frailty itself…”*

- What does the term ‘frailty’ mean to you?
- What signs or symptoms would a person have to have for you to consider them as frail?
  - **Prompt:** How would you describe a frail person?
  - **Prompt:** Other than physical signs what other signs would you associate with a frail person?
  - **Prompt:** Social? Psychological? Cognitive?
- Do you think frailty is an inevitable consequence of ageing?
  - **Prompt:** Why do you think this?

*“Currently there is no single agreed clinical definition of frailty. I would like to talk about frailty using this definition; can I ask you to read this please?*

[Hand participant a card with definition printed on it]

***Frailty is a reduction in functioning across multiple physiological systems, which heightens an individual’s vulnerability to stressors.”***

*A frail older person could experience multiple deficits across one or more areas such as* ***physical,*** ***psychological, social, and cognitive****, leading to different symptoms including unexpected weight loss, slow gait speed, cognitive impairment, mood disorders, loneliness, multiple co-morbidities, or medications depending on the severity of their frailty.*

*The severity of an individual’s frailty can be classed in multiple different ways depending on the (assessment) tool being used. The electronic frailty index currently used by GPs in practice diagnoses patients as fit (not frail) or as having mild, moderate, or severe frailty depending on the number of symptoms or deficits experienced by the individual. A person’s frailty status can improve or worsen over a period of time.”*

*“For the purposes of this study we’re focusing on older people (those aged 65 years and over) with frailty, still living in their own homes as opposed to a nursing or residential home, so please bear this in mind as you answer the remainder of the questions.”*

- Approximately what proportion of older (≥65 years) patients in this pharmacy would you consider to be frail?
- On a typical working day in this pharmacy, approximately how many older people with frailty would you encounter (e.g. face-to-face or over the telephone)?
- Do you think it is important for community pharmacists to know a patient’s frailty status?
  - **Prompt:** Why?
  - **Prompt:** How would knowledge of a patient’s frailty status influence how you provide pharmaceutical care for that patient?
- Do you assess older patients for frailty as part of your routine practice?

| **If participant answers ‘Yes’** | **If participant answers ‘No’** |
| --- | --- |
| - Why do you assess people for frailty?   - - **Prompt:** What is it about these patients that makes you assess them for frailty? - How do you go about assessing a patient for frailty?   - - **Prompt:** Are there any diagnostic tools or tests that you use to identify frailty?     - **Prompt:** Can you tell me more about these? - What would prevent you from assessing a person for frailty?   - - **Prompt:** If assessing patients involved physical examinations would that be prohibitive? - What would help you in assessing a person’s frailty status? | - Why? - Are you aware of how you might go about assessing a patient for frailty?   - - **Prompt:** Do you know of any diagnostic tools or tests that can be used to identify frailty?     - **Prompt:** Can you tell me more about these? - What would prevent you from assessing a person for frailty?   - - **Prompt:** If assessing patients involved physical examinations would that be prohibitive? - What would help you in assessing a person’s frailty status? |

- Have you ever discussed a patient’s frailty status with another healthcare professional?
  - - **Prompt:** Why?
    - **Prompt:** Who initiated the discussion?
    - **Prompt:** Did knowledge of the patient’s frailty status alter their treatment or care plan? What was the end result and was it beneficial for the patient?
- Have you ever discussed a patient’s frailty status with them?
  - - **Prompt:** Why?
    - **Prompt:** Who initiated the discussion? Or What prompted the discussion?
    - **Prompt:** How did the patient react to or perceive the diagnosis of frailty?
    - **Prompt:** What was the end result and was it beneficial for the patient?

**Medication-related issues in frail older people**

*“I’d now like to focus on medicines use by frail older people, and how medicines can be optimised for these patients. The term ‘medicines optimisation’ has now superseded older terms such as ‘pharmaceutical care’ and ‘medicines management’. The National Institute for Health and Care Excellence (NICE) defines medicines optimisation in the following way:*

[Hand interview participant a card with definition printed on it]

*‘Medicines optimisation is a person-centred approach to safe and effective medicines use to ensure that people gain the best possible outcomes from their medicines. It also takes a multidisciplinary approach with the aim of improving all aspects of patient-centred care. This then includes the key components of* ***medicine selection, medicine delivery, prescribing and deprescribing choices, medicine administration, patient knowledge and adherence, and medication review****. All to ensure a patient is receiving the maximum benefit from their medication while minimising the risk of adverse effects and any possible harm.’*

*“I am now going to show you an example of an older frail patient and the medicines they receive. This scenario is not intended as a test. I would like you to take a few minutes to review the patient’s medication list. When you are ready, I’d like to ask you a few questions about your main concerns with their medicines and the key issues that you would prioritise if you were to attempt to make any changes to the patient’s prescription.*

*Does that sound OK to you?*

*Take your time to look through this list and when you are ready, I will ask you some questions.”*

[Hand interview participant a card with clinical scenario printed on it]

**Esme Shaw**

Esme is an 85-year-old retired shop owner. She is recently widowed, and currently lives alone in her home with her dog Walter. She has no family living close by. Esme is mobile at home with the assistance of a walker and is able to prepare her own meals and make herself a hot drink.

- Esme was previously assessed as being ***mildly frail.***

Recently neighbours have noticed a reduction in Esme’s walking speed and the time taken to get up and out of a chair. Esme is no longer able to leave the house for her usual outside activities (WI, shopping, doctors’ appointments) without support.

Esme is currently on medication for a number of chronic conditions including hypertension, Type 2 diabetes mellitus and osteoporosis, all of which is dispensed from her local community pharmacy.

- During her last visit to her GP, Esme was assessed as being ***moderately frail*** according to the electronic frailty index***.***

**Medication list**

| ALENDRONATE 70mg tablets | Take ONE weekly |
| --- | --- |
| COLECALCIFEROL 800u tablets | Take ONE mane |
| METFORMIN 1g tablets | Take ONE bd |
| RAMIPRIL 7.5mg capsules | Take ONE mane |
| DIAZEPAM 2mg tablets | Take ONE tid for anxiety when needed |
| DOSULEPIN 75mg tablets | Take ONE daily |
| OXYBUTYNIN 5mg tablets | Take ONE tid |
| OMEPRAZOLE 20mg capsules | Take ONE daily |
| SALBUTAMOL 100mcg dose inhaler | Take ONE-TWO puffs when required |
| CLENIL 200mcg dose inhaler | Take TWO puffs twice daily |

- Does Esme fairly represent a frail older patient that you could see in your pharmacy?
  - **Prompt:** What about Esme would indicate a diagnosis of frailty?
    - **Prompt:** Background? Medication?
- Could you tell me briefly, what are the main issues that you would be concerned about in this patient?
  - **Prompt:** How would you go about addressing this? Who would you contact first? What, if anything, would you say to the patient?
- If you were to attempt to address and resolve these issues, what would you prioritise and why?
- Are there any other medication-related issues you have experienced with frail older people that you would like to tell me about?
  - **Prompt:** How did you go about addressing this? Who did you contact first? What, if anything, did you say to the patient?
  - **Prompt:** What happened as a result of you acting?
  - **Prompt:** Do you feel these issues were specific to older **frail** patients?
- What role do you currently play as a community pharmacist for frail older people?
  - **Prompt:** If you don’t feel you currently play a role, then what role do you think you should play?
- What services do you currently provide in your pharmacy that you could offer to a patient like Esme?
  - - **Prompt:** Do you think that older patients with frailty use more pharmacy services than those without frailty?
- Are the current community pharmacy services sufficient for frail older people like Esme living in their own homes?
  - - **Prompt:** Why?
    - **Prompt:** What other services do you think would be beneficial?
    - **Prompt:** Do older frail patients have different needs than other patients?
- What services or organisations could you sign-post Esme to for additional help and support?
  - - **Prompt:** Have you ever referred, or sign-posted a patient to a service or organisation for support because of their frailty status?

**Prompt:** Why did you refer them and what was the outcome?

- - **Prompt:** Do you feel signposting patients to these services is relevant to your role as a pharmacist?

**Future role in optimising medicines use by frail older people**

*“The team undertaking this research study are interested in interventions or approaches to optimise medicines use by frail older people in primary care. From our research so far, we know that there are a limited number of approaches that have been developed which focus on optimising medicines use by frail older people. I would like you to think about how community pharmacists could contribute to such interventions.”*

- What could community pharmacists do differently to optimise medicines use for frail older people in primary care?
- What things would help you in doing this?
- What things would hinder you in doing this?
- Should anyone else be involved in helping to optimise medicines for frail older people in primary care?
  - **Prompt:** GPs, practice nurses, practice pharmacists, carers

**Closing the interview**

*“That brings us to the end of the interview.*

*Is there anything else about medicines optimisation in older people with frailty that you feel has not been covered?*

*Do you have any additional comments you would like to make about the content of the interview or how it went?*

*Thank you so much for making the time to speak with me today.”*

[Turn the digital recorder off]
